# Chronic Disease Self-Management Education (CDSME) Program Delivery and Attendance among Urban-Dwelling African Americans

**DOI:** 10.3389/fpubh.2014.00174

**Published:** 2015-04-27

**Authors:** Chivon A. Mingo, Matthew Lee Smith, SangNam Ahn, Luohua Jiang, Jinmyoung Cho, Samuel D. Towne, Marcia G. Ory

**Affiliations:** ^1^Gerontology Institute, College of Arts and Sciences, Georgia State University, Atlanta, GA, USA; ^2^Department of Health Promotion and Behavior, College of Public Health, The University of Georgia, Athens, GA, USA; ^3^Division of Health Systems Management and Policy, School of Public Health, The University of Memphis, Memphis, TN, USA; ^4^Department of Health Promotion and Community Health Sciences, School of Public Health, Texas A&M Health Science Center, College Station, TX, USA; ^5^Department of Epidemiology and Biostatistics, School of Public Health, Texas A&M Health Science Center, College Station, TX, USA; ^6^Baylor Scott & White Health, Center for Applied Health Research, Temple, TX, USA

**Keywords:** African American, urban, chronic disease self-management, delivery site, evidence-based program

## Abstract

**Background:**

Older African Americans carry a disproportionate share of chronic diseases. The purpose of this study was to identify the characteristics of urban-dwelling African Americans with chronic disease participating in Chronic Disease Self-Management Education (CDSME) programs and to examine factors related to successful program completion (i.e., attending at least four of the six sessions).

**Methods:**

Data were analyzed from 11,895 African Americans who attended a CDSME program at one of the five leading delivery sites (i.e., senior center, health care organization, residential facility, community location, faith-based organization). Logistic regression analyses were used to assess the associations of demographic, delivery site, and neighborhood characteristics with CDSME program successful completion.

**Results:**

Approximately, half of the African American participants were aged 65–79 years, 83% were female, and 92% lived alone. Approximately, 44% of participants had three or more chronic conditions and 35% resided in an impoverished area (i.e., 200% below federal poverty level). Successful completion of the CDSME program was associated with being between the ages of 50–64 and 65–79 years, being female, living alone, living in an impoverished community, and attending a CDSME program at a residential facility or community center.

**Conclusion:**

Findings highlight the unique patterns of attendance and delivery within the context of self-management interventions among this unique and traditionally underserved target population. Understanding such patterns can inform policy and practice efforts to engage more organizations in urban areas to increase CDSME program adoption. Particularly, employing strategies to implement CDSME programs across all delivery site types may increase reach to African American participants.

## Introduction

Currently, over 43 million US residents are 65+ years of age with that number projected to increase to approximately 80 million by the year 2040 (1). Along with the overall growth of the aging population, a significant increase in the racial/ethnic diversity of this population is also occurring. Racial/ethnic minority populations are projected to increase to 20.2 million making up 28% of the aging population in 2030 (1). Specifically, between the years of 2012 and 2030, it is projected that the non-Hispanic African American population aged 65 years and above will increase 104% in comparison to the 54% projected increase for non-Hispanic Whites in the same age group (1).

The drastic increase in the number of older adults, particularly those from diverse racial/ethnic groups, is associated with burgeoning rates of chronic disease. Approximately, 50% of aging adults report two or more chronic conditions, and data show that the prevalence of having two or more comorbidities is higher among aging African Americans than other racial/ethnic groups (i.e., Whites, Hispanics) (2). Moreover, African Americans are more likely to be diagnosed with a chronic condition at a younger age and be more physically disabled than Whites (3). Although African Americans are at a greater risk for a chronic disease diagnosis and negative health outcomes associated with chronic conditions in comparison to Whites, African Americans are underrepresented in access to chronic disease self-management education programs as a result of a variety of biopsychosocial and sociocultural factors (e.g., racism, low socioeconomic status, discrimination, unequal access to goods and services, lack of trust in the health care system) (4–6). It is plausible that the context in which one experiences negative health outcomes may impact their perceptions about health, health behaviors, and disease management (7–9). However, very little attention has been given to cultural milieu and unique experiences for African Americans that may influence utilization, participation, and even completion of evidenced-based health prevention and management programs [e.g., Chronic Disease Self-Management Program (CDSMP)].

Considerable efforts have been made nationally to address issues surrounding the management of chronic illnesses among underserved and disadvantaged individuals (10–13). In fact, public health efforts have focused on reducing the burden of chronic disease for all and minimizing health disparities in chronic disease among racial/ethnic minorities by redirecting society’s attention to the benefits of evidence-based health prevention and management programs. Notably, there has been a concerted effort by the US Administration on Aging, Centers for Disease Control and Prevention (CDC), and Centers for Medicare and Medicaid Services (CMS) to disseminate and implement Chronic Disease Self-Management Education (CDSME) programs nationally (14).

As a result, a national study of the CDSMP was conducted resulting in greater racial/ethnic diversity among participants than had been seen in previous research focused on self-management (10, 11, 13). Specifically, the national study included approximately 45% of participants who self-identified as African American, Latino, or other minority groups (11). Although substantial efforts to widely disseminate and implement the program have been made, there is still a great deal of information to be learned about how individuals living with multiple chronic conditions (MCC) successfully manage their conditions by participating in and completing evidenced-based self-management programs (e.g., CDSMP) and how this may vary by race/ethnicity.

While much has been written about national dissemination and implementation of the CDSME programs (10–12) as well as the benefits of completing a CDSME program (13), there is little empirical data that examine the effectiveness of the program or the unique predictors of participation and successful completion among African Americans with chronic conditions. Moreover, few studies have reported on completers (i.e., individuals who complete four out of six sessions) and non-completers (i.e., individuals who stop attending the program prior to completing four sessions) within this context. Thus, the fact that African Americans are already disproportionately affected by chronic conditions is made worse by the relative gap in what is known about the management of chronic diseases among this target population.

In addition to African Americans facing unique challenges (e.g., racism, discrimination, cultural mistrust) that may influence health behaviors, health status, and utilization of health care (4–6), African Americans living in urban areas may add another layer of complexity that is not well addressed or understood in the chronic disease self-management literature. African Americans who reside in urban areas often experience unique health-related vulnerabilities in comparison to Whites and other minority racial/groups. A significant proportion of African Americans reside in urban areas overwhelmed with violence, dilapidated housing, limited options for fresh fruits and vegetables, and in close proximity to toxic waste sites (15–17). Further, research has shown that specifically urban-dwelling aging African Americans have greater levels of disability as a result of chronic conditions in comparison to not only Whites but also aging African Americans living outside of urban areas (18, 19).

The aforementioned findings suggest the importance of extending CDSME research in an effort to understand the patterns of chronic disease management among urban-dwelling aging African Americans. Studying African Americans specifically in this context is a unique contribution to the literature in that African Americans embody cultural similarities but also intragroup differences. It is important for researchers, health care providers, and policy makers to recognize the vast array of characteristics that may be represented in aging African Americans in order to provide services that are appealing, feasible, and that lead to successful completion of effective behavioral health programs.

Therefore, the objective of this study is to (1) determine the characteristics of urban-dwelling African Americans with at least one chronic condition who participated in a CDSME program, and (2) determine factors associated with successfully completing the program.

## Materials and Methods

### Data source and study population

Cross-sectional data for this study was obtained from a nationwide delivery of CDSME programs as part of the American Recovery and Reinvestment Act of 2009 (i.e., Recovery Act) *Communities Putting Prevention to Work: Chronic Disease Self-Management Program* initiative (20). The US Administration on Aging led this initiative in collaboration with CDC and CMS to support the translation of CDSME programs in 45 states, Puerto Rico, and the District of Columbia (14). This initiative was originally designed to have 50,000 Americans complete CDSME program sessions between 2010 and 2012 and to embed CDSME program delivery structures into statewide systems (20). Based on the aforementioned unique challenges facing the target population, the focus of this study is on the 11,895 urban-dwelling African Americans who attended the program at one of the five leading delivery sites (i.e., senior center, health care organization, residential facility, community location, faith-based organization) and reported having at least one chronic condition. Specifically, 2,170 unique workshops were delivered to African American participants in this study. Of these workshops, 754 were delivered in senior centers/AAA, 518 in healthcare organizations, 479 in residential facilities, 242 in community/multi-purpose facilities, and 177 in faith-based organizations. Participants attending other sites were small in sample size and omitted from the analyses in this study. Institutional Review Board approval for this study was given by Texas A&M University.

### Program description

The CDSMP, a program included within a larger suite of Stanford’s CDSME programs, has been introduced and widely disseminated in the US as a method to empower patients with self-management skills to deal with their chronic conditions (21). Drawing upon Social Learning Theory (22), CDSME programs are evidence-based, peer-led interventions consisting of six highly participative classes held for 2.5 hours each, once a week, for six consecutive weeks (21). During the tenure of these programs, participants receive a copy of the book, *Living a Healthy Life with Chronic Conditions, Fourth Edition* (23) as well as an audio relaxation CD titled *Relaxation for Mind and Body* (24). In addition, content for the workshops focus on teaching individuals ways to deal with frustration, isolation, pain, and fatigue. Furthermore, participants are taught how to develop an action plan to meet intended goals; how to develop an individualized exercise program; how to appropriately use medications; how to solve chronic disease-related problems; and how to communicate with family, friends, and health care providers. In particular, the CDSMP has resulted in improved health care (e.g., exercise, communication with physician) and health (e.g., pain, self-reported health, fatigue, disability, depression) (11–13), while potentially saving health care costs (25).

### Measures

#### Independent variables

In an effort to identify characteristics associated with participating and successfully completing the CDSME program that are specific to urban-dwelling African Americans with chronic conditions, various measures (i.e., demographics, health status, delivery site types) were included.

##### Demographics

Participants were asked to self-report age (i.e., in years), sex (i.e., male or female), and living arrangement (i.e., living alone, living with others). In addition, residential ZIP Codes provided by participants were used to determine percentage of residents within the ZIP Code that fell below a 200% federal poverty level, and the percent of African American residents residing within the participants’ ZIP Code.

##### Health status

Health status was determined using the participants’ self-reported chronic conditions. Participants were presented with a list of chronic conditions (i.e., arthritis, cancer, depression, diabetes, heart disease, hypertension, lung disease, stroke, osteoporosis, other) and asked to indicate whether or not they had been diagnosed with each condition by a health care provider. The number of self-reported chronic conditions were summed to create one count variable, and the prevalence of each individual disease was calculated based on participants’ self-report.

##### Delivery site type

Delivery site information was collected through administrative procedures. For the purposes of this study, analyses focused on the five leading delivery sites (i.e., senior center, health care organization, residential facility, community location, faith-based organization). Participants attending CDSME programs at any other delivery site types (e.g., workplaces, educational institutions, tribal centers) were omitted because of inadequate case sizes. Reports of participants attending the CDSME program at any delivery sites or delivery sites labeled as “other” were omitted from analyses due to the complexities of interpretation.

#### Dependent variable

##### Successful completion

Completion of the CDSME program was the dependent variable of interest in this study. Attending at least four of the six classes was considered successful completion of the program. This criterion is consistent with criterion used in previous work focused on the CDSMP (11, 12, 20).

### Statistical analysis

First, frequencies were examined to assess demographics, health status, and utilization by delivery site type among the total sample of urban-dwelling African Americans with chronic disease. Next, independent samples *t*-test, one-way ANOVA, and chi-square analyses were conducted to examine differences in sample characteristics by number of chronic conditions (i.e., one, two, or three or more chronic conditions) and by CDSME programs completion status (i.e., non-successful completion, successful completion). Specifically, independent samples *t*-test analyses and one-way ANOVA were used for continuous variables and chi-square analyses were used for categorical variables. Subsequently, a multiple logistic regression model was employed to determine the association between the independent variables (i.e., age, sex, living situation, number of chronic conditions, delivery site, poverty level) and successful completion of CDSME programs.

## Results

Descriptive statistics for the sample of urban-dwelling African Americans (*N* = 11,895) are displayed in Table 1. Descriptive statistics are presented for the total sample and stratified by the number of self-reported chronic conditions and CDSME program completion status. On average, participants were 68 (±12.06) years of age. The majority of the participants were female (83%) and lived with someone (92%). On average, participants reported having 2.5 (±1.41) chronic conditions with approximately 44% reporting 3 or more. Arthritis (50%), diabetes (48%), and hypertension (64%) were the three most commonly reported chronic conditions among the total sample. The largest percentage of participants attended the CDSME program at a senior center (37%) or residential facility (24%). Thirty-five percent of our sample resided in an impoverished area (i.e., 200% below federal poverty level).

**Table 1:** **Sample characteristics by number of chronic conditions and CDSME program completion**.

	Total (*n* = 11,895)	1 Chronic condition (*n* = 3,331)	2 Chronic conditions (*n* = 3,294)	3+ Chronic conditions (*n* = 5,270)	*X*^2^ or *f*	*p*	Non-successful completion (*n* = 2,445)	Successful completion (*n* = 9,450)	*X*^2^ or *t*	*p*
Age (years)	68.46 (±12.06)	66.95 (±13.70)	68.46 (±12.21)	69.41 (±10.69)	42.57	<0.001	67.21 (±12.69)	68.78 (11.87)	−5.53	<0.001
Under 50	6.6%	10.8%	6.9%	3.7%	180.53	<0.001	8.4%	6.1%	35.89	<0.001
50–64	26.5%	25.5%	27.2%	26.8%			29.4%	25.8%		
65–79	49.7%	46.6%	47.8%	53.0%			46.0%	50.7%		
80+	17.1%	17.2%	18.1%	16.5%			16.2%	17.4%		
Sex
Male	16.8%	22.1%	16.0%	13.9%	100.52	<0.001	19.6%	16.1%	16.69	<0.001
Female	83.2%	77.9%	84.0%	86.1%			80.4%	83.9%		
Lives alone
No	92.3%	79.3%	97.3%	97.5%	1115.18	<0.001	96.4%	91.3%	70.28	<0.001
Yes	7.7%	20.7%	2.7%	2.5%			3.6%	8.7%		
Number of chronic conditions	2.53 (±1.41)	–	–	–	–	–	2.64 (±1.47)	2.51 (±1.40)	3.93	<0.001
1	28.0%	–	–	–	–	–	25.8%	28.6%	8.42	0.015
2	27.7%	–	–	–			27.8%	27.7%		
3+	44.3%	–	–	–			46.4%	43.8%		
Disease prevalence
Arthritis	50.3%	14.9%	44.7%	76.3%	3139.78	<0.001	52.1%	49.9%	3.98	0.046
Cancer	7.4%	2.0%	4.9%	12.4%	369.93	<0.001	7.9%	7.3%	1.21	0.271
Depression	14.4%	3.1%	8.2%	25.3%	960.76	<0.001	18.1%	13.4%	35.38	<0.001
Diabetes	47.9%	34.8%	38.4%	62.1%	777.04	<0.001	44.2%	48.9%	16.87	<0.001
Heart disease	17.5%	2.3%	9.5%	32.2%	1465.08	<0.001	18.5%	17.3%	2.05	0.152
Hypertension	64.1%	26.3%	66.2%	86.6%	3234.57	<0.001	65.1%	63.8%	1.37	0.242
Lung disease	18.9%	3.9%	8.1%	35.2%	1651.38	<0.001	21.7%	18.2%	15.30	<0.001
Stroke	7.7%	1.3%	4.3%	13.9%	530.15	<0.001	8.6%	7.5%	3.67	0.055
Osteoporosis	10.0%	1.6%	5.3%	18.1%	729.44	<0.001	10.4%	9.8%	0.78	0.378
Other	15.0%	9.9%	10.4%	21.1%	276.60	<0.001	16.8%	14.6%	7.27	0.007
Percent of ZIP Code under 200% poverty level	34.59 (±8.22)	35.14 (±8.02)	34.33 (±8.21)	34.40 (±8.34)	10.73	<0.001	34.72 (±8.15)	34.55 (±8.24)	0.89	0.375
Percent of African American residents in ZIP Code	47.48 (±29.20)	47.80 (±29.49)	47.81 (±29.22)	47.06 (±29.01)	0.96	0.381	48.67 (±27.70)	47.17 (±29.07)	2.24	0.025
Delivery site type
Senior Center/AAA	36.5%	40.0%	34.5%	35.6%	178.72	<0.001	31.2%	37.9%	82.70%	<0.001
Healthcare organization	15.5%	13.7%	16.6%	15.8%			18.0%	14.8%		
Residential facility	24.1%	18.6%	22.7%	28.4%			29.4%	22.7%		
Community/multipurpose-facility/library	11.2%	11.6%	12.5%	10.0%			10.6%	11.3%		
Faith-based organization	12.8%	16.0%	13.7%	10.2%			10.8%	13.3%		

When examining differences in characteristics across number of chronic conditions (i.e., one chronic condition, two chronic conditions, three or more chronic conditions), a statistically significant difference was found among the three groups on age, sex, living situation, disease prevalence, poverty level, and delivery site type. Notably, larger proportions of participants reporting three or more chronic conditions were ages 65–79 years (53%), female (86%), and living with someone (98%). As expected, larger proportions of participants who reported having any of the chronic conditions ultimately had more comorbidities. A larger proportion of participants who attended workshops at senior centers and faith-based organizations (40%, 16%; respectively) reported only one chronic condition. Conversely, a larger proportion of participants who attended a workshop at a health care organization or community center (17%, 13%; respectively) reported two chronic conditions. Lastly, a larger proportion of participants who attended the CDSME program at a residential facility (28%) reported three or more chronic conditions.

Table 1 also reports differences in completion status across all independent variables. Significant differences in non-successful completion/successful completion were found across age, sex, living situation, number of chronic conditions, prevalence of disease by disease type, and type of delivery site. A larger proportion of participants who successfully completed the CDSME program were older (*p* < 0.001), female (*p* < 0.001), report living alone (*p* < 0.001), and report fewer chronic conditions (*p* < 0.001). A significantly smaller proportion of successful completers reported arthritis (*p* = 0.05), depression (*p* < 0.001), lung disease (*p* < 0.001), whereas a larger proportion of successful completers reported having diabetes (*p* < 0.001). There were no significant differences found in cancer, heart disease, hypertension, stroke, and osteoporosis disease prevalence between successful and non-successful completers. Lastly, significant differences were found across delivery sites between those who successfully completed CDSME programs and those who did not. A larger proportion of participants who successfully completed CDSME programs attended senior centers, whereas a smaller proportion of successful completers attended CDSME programs at other delivery sites (i.e., health care organization, residential facility, community center, faith-based organization) (*p* < 0.001).

Table 2 provides results of the logistic regression analyses. Compared to participants below the age of 50 years, being between the ages of 50–64 and 65–79 years decreased the odds of successfully completing the CDSME program [odds ratio (OR) = 0.71, *p* = 0.001 and OR = 0.85, *p* = 0.025; respectively). However, the odds of successfully completing the program increased for females (OR = 1.24, *p* < 0.001) and those that live alone (OR = 2.38, *p* < 0.001). Number of chronic conditions was not significantly associated with successful completion. Compared to senior centers, attending the CDSME program at a residential facility or a community center decreased the odds of successfully completing the program (OR = 0.70, *p* < 0.001; OR = 0.61, *p* < 0.001). In addition, living in an impoverished neighborhood reduced the odds of successfully completing the CDSME program (OR = 0.99, *p* < 0.024).

**Table 2:** **Factors associated with successful completion**.

	95% CI			
	OR	*P*	Lower	Upper
Age: under 50 years	1.00	–	–	–
Age: 50–64 years	0.71	0.001	0.578	0.863
Age: 65–79 years	0.85	0.025	0.738	0.980
Age 80+ years	1.02	0.763	0.897	1.160
Male	1.00	–	–	–
Female	1.24	<0.001	1.106	1.397
Live alone: no	1.00	–	–	–
Live alone yes	2.38	<0.001	1.892	3.007
Number of chronic conditions: 1	1.00	–	–	–
Number of chronic conditions: 2	1.04	0.454	0.932	1.172
Number of chronic conditions: 3+	1.05	0.365	0.944	1.171
Senior Center/AAA	1.00	–	–	–
Healthcare organization	0.92	0.280	0.785	1.073
Residential facility	0.70	<0.001	0.590	0.833
Community/multipurpose facility/library	0.61	<0.001	0.523	0.720
Faith-based organization	0.87	0.155	0.719	1.054
Percent of ZIP Code under 200% poverty level	0.99	0.024	0.988	0.999

*OR, odds ratio; 95% CI, 95% confidence interval; attending fewer than four workshop sessions, referent*.

## Discussion

### Identification of similarities and differences

An initial research objective was to identify the characteristics of African American participants and the program delivery infrastructure that served them. In many regards, the urban-dwelling African American participants with chronic disease had profiles similar to participants seen in earlier CDSMP studies (11, 20, 26, 27). For example, participants were older, predominantly female, reported their most prevalent chronic conditions as hypertension, arthritis, and diabetes, and were more likely to participate in the program at senior centers, residential facilities and health care organizations. Notably, in our sample participating in CDSME programs at a residential facility was more prevalent than participating at a health care organization; the order of prevalence was reverse in the overall national level sample (20, 27).

Although the participants in our study were demographically similar in many ways to those in previous research describing populations and delivery characteristics, this study clearly demonstrates unique characteristics that are worthy of highlighting, and important to consider in policy, practice, and future research. Particularly, the burden of chronic disease among urban-dwelling African Americans cannot be ignored. While the ranking of chronic condition prevalence rates for specific disease type was similar to CDSME program participants in other studies (20), the actual prevalence rates for the individual chronic conditions were substantially higher. National prevalence rates indicate that irrespective of racial/ethnic group 21% of US community-dwelling adults report having three chronic conditions and approximately 5% report four or more (28). Overall, 44% of our sample reported having three or more chronic conditions. Moreover, our research findings indicated that participants with different comorbidity levels (i.e., one, two, three or more) participated in the CDSME program at different delivery site types. Specifically, larger proportions of participants who attended the CDSME at senior centers and faith-based organizations reported only having one chronic condition, whereas a larger proportion of those participating at a residential facility reported three or more chronic conditions. This is not overwhelmingly surprising in that residential facilities may be servicing individuals living within their community. Oftentimes, decisions to move into residential facilities particularly for aging individuals is predicated on disabilities and health challenges associated with common chronic conditions (29).

### Associations with program completeness

In addition to examining differences across chronic conditions, our study also closely examined differences in characteristics for successful completers and non-completers of the program as well as identified factors that were associated with successful completion. Results indicated that successful completers of the program were older than those who did not complete the program. However, the regression analyses showed that compared to participants below the age of 50 years, being between the ages of 50–64 and 65–69 years decreased the odds of successfully completing CDSME programs.

Considering that African Americans are diagnosed with chronic conditions much earlier in life in comparison to other groups (6), it may be that those between the age of 50–64 and 65–79 years have been dealing with the condition(s) for a longer period of time and either feel that they have learned how to successfully manage their health or have a perception that nothing can really be done for their condition. For example, in a study examining perceptions of arthritis, the authors found that individuals were less likely to believe a person diagnosed with arthritis could improve their condition with better health care (30). Perceptions of this type would make one more vulnerable to not completing the program. Studies have shown that the perception of chronic conditions and symptoms associated with chronic conditions influences health care decisions among African Americans (30, 31). In addition to age, a larger proportion of females and those living alone were successful completers. Moreover, being female and living alone increased the odds of successfully completing the CDSME program. Our findings on gender differences are consistent with findings in previous research (26, 32).

### Implications for reaching males

Due to the limited number of African American male participants and the findings highlighting gender differences in completion status, it is important to note the implications for the health of African American males. Unfortunately, the health of African American males has been likened to that of individuals living in developing countries (33). African American males fare worse than other segments of the population (e.g., African American females, Whites, and other racial/ethnic minority groups) on almost every chronic condition (33). While there is a dismal focus on African American men in chronic disease self-management research (13), our findings suggest that it is paramount that emphasis is put on empowering urban-dwelling African American men to engage in health promotion that would ultimately lead to positive health outcomes and providing other health-related benefits (e.g., symptom management, reduction of health care cost, reduction in emergency room visits, reduction in work disability) at both the individual and societal level.

### Associations with chronic conditions

When examining health status in this study, individuals who completed the CDSME program had fewer total number of chronic conditions and reported lower prevalence rates of arthritis, depression, and lung disease. Interestingly, successful completers in comparison to non-completers reported higher rates of diabetes and no significant differences for the other conditions. Moreover, the total number of chronic conditions was not associated with successful completion. Erdem and Korda (26) reported similar findings in a study that examined characteristics of participants with diabetes who completed both the CDSMP and Diabetes Self-Management Program (DSMP). Future research is warranted to further examine the impact of MCC on participation and completion of behavioral health interventions.

### Associations with environmental characteristics

While there were no significant differences in successful completers and non-completers based on neighborhood level poverty, living in an impoverished community was associated with a lower likelihood of successfully completing the program. Research has consistently documented the association between socioeconomic factors and health, particularly highlighting the vulnerabilities of living in poverty (34). It is plausible that living in areas of concentrated poverty introduces additional barriers that may interfere with not only participating but completing health programs that are beneficial. As previously stated, a significant proportion of urban-dwelling African Americans live in impoverished areas that are associated with violence, poor housing, and limited access and availability to options that promote health (15–17).

To our knowledge, no research has closely examined the association of living in an impoverished community with completing the CDSME program among urban-dwelling African Americans. Findings not only suggest the need for future research in this area but also provide preliminary results that should be considered when implementing and disseminating the CDSME program in certain communities. Providing additional support that would foster increased completion rates of the CDSME program to residents of this community could lead to positive health outcomes for a population that is at risk for continued health and health care disparities.

### Associations with delivery site

Finally, our analyses indicated that delivery site type is an important factor for urban-dwelling African Americans when considering program completion. Senior centers had the largest proportion of completers whereas faith-based organizations and community centers had the smallest proportion of completers. However, it is important to note among African Americans only that faith-based organizations and community centers had the smallest proportion of program participants. When examining the association between delivery sites and successful completion, findings indicated that the odds of successfully completing the program were significantly decreased if one attended the CDSME program at a residential facility or community center compared to attending the program at a senior center. Interestingly, there was no significant relationship between successful completion and faith-based organizations or health care organizations. This is in contrast to another study including participants from multiple racial/ethnic groups that found that completion rates for the CDSME program are lowest at residential facilities but highest at faith-based organizations (26). Other studies focused on delivery preferences of a self-management program among aging individuals who lived in an urban area have found that, in comparison to Whites, African Americans were more likely to prefer a self-management program that would be delivered at a local church or a health care organization (35). Notably, implementing programs within faith-based organizations have shown to be effective in increasing utilization of health promotion programs for African Americans (36–41). Therefore, the finding that faith-based and health care organizations have no association with successful completion may be more about reach and less about preference for participating or completing at that site type.

Therefore, one promising strategy for increasing the reach of CDSME programs may be to work more closely with faith-based organizations and health care organizations in urban African American communities. Embedding the program in existing infrastructures (e.g., church health ministry, senior ministry) may yield greater participation and completion of the program. Findings indicating that the odds of successfully completing the program are lowered for those participating at a residential facility or at a community center may be a result of a number of barriers. For example, residential facility participants may have a number of health complications resulting in the inability to successfully complete the program. Individuals participating in CDSME programs at a local community center may have other family responsibilities or transportation issues that could serve as a barrier to participating in a program for six consecutive weeks.

### Study limitations

Given that many of the measures were collected using self-reports, it is possible that participants may over- or underreport health status variables. However, self-report of chronic conditions is used as a valid measure in studies examining health in aging individuals (42). In addition, the cross-sectional nature of the data does not allow for us to determine a causal relationship between the independent variables and outcome variables. Although the delivery site type is available, lack of information concerning how participants were recruited to participate in the program is unavailable. It may not just be the differences in the types of delivery sites that result in significant differences in successful completion of the CDSME program, but also the methods that delivery sites use to initially recruit participants in the program. While lay leaders are trained to offer the program in a standardized manner, another limitation to consider would be unaccounted variance in program implementation that may or may not impact participants to remain in the program. Lastly, limited information is available about the lay leaders who served as instructors for the workshops. Therefore, an additional limitation of the study is the inability to examine the impact of instructor-level factors on completion rates (e.g., race, gender, age, health status).

## Conclusion

The *American Recovery and Reinvestment Act Communities Putting Prevention to Work: Chronic Disease Self-Management Program* (14) has made a large public impact on the ongoing national dissemination and implementation of CDSME programs. The present study yields findings that can contribute to the ongoing research, practice, and policy efforts associated with this initiative. Particularly, our findings indicate that for vulnerable populations such as urban-dwelling African Americans, the influence of the individual, social, and environmental context in which one may experience CDSME programs must be considered. Strategies to encourage employment of CDSME programs across all available delivery site types can foster participation and completion. Working closely with health care providers and community gatekeepers to inform individuals about the availability and benefits of completing CDSME programs is one method for moving forward. In addition, considerations for program modifications that would still yield similar outcomes, but foster greater levels of completion should be discussed. Also, it is likely that putting policies in place that allow for allocation of resources that would provide support to individuals in impoverished communities may also yield positive outcomes. In summary, our study indicates that in order to increase reach and positively impact, a diverse population, practice, policy, and research strategies must consider the cultural milieu for African Americans that ultimately influence chronic disease self-management.

## Conflict of Interest Statement

The authors declare that the research was conducted in the absence of any commercial or financial relationships that could be construed as a potential conflict of interest.

This paper is included in the Research Topic, “Evidence-Based Programming for Older Adults.” This Research Topic received partial funding from multiple government and private organizations/agencies; however, the views, findings, and conclusions in these articles are those of the authors and do not necessarily represent the official position of these organizations/agencies. All papers published in the Research Topic received peer review from members of the Frontiers in Public Health (Public Health Education and Promotion section) panel of Review Editors. Because this Research Topic represents work closely associated with a nationwide evidence-based movement in the US, many of the authors and/or Review Editors may have worked together previously in some fashion. Review Editors were purposively selected based on their expertise with evaluation and/or evidence-based programming for older adults. Review Editors were independent of named authors on any given article published in this volume.
